# Relationships between dimensional factors of psychopathy and schizotypy

**DOI:** 10.3389/fpsyg.2013.00482

**Published:** 2013-07-26

**Authors:** Katie A. Ragsdale, Jeffrey S. Bedwell

**Affiliations:** Department of Psychology, University of Central FloridaOrlando, FL, USA

**Keywords:** psychopathy, schizotypy, self-centered impulsivity, fearless dominance, schizophrenia

## Abstract

Existing research has suggested that comorbid psychopathy may explain one trajectory of violent behavior in a subset of individuals with schizophrenia. However, it remains unclear which specific traits and symptoms are responsible for this relationship and whether it is limited to clinical and/or forensic categories, or if it reflects a dimensional relationship found in the general population. Therefore, the aim of this study was to examine differential relationships between specific factors of psychopathy and schizotypy in a non-psychiatric and non-forensic sample. Two hundred and twelve undergraduate students (50% female) completed the Schizotypal Personality Questionnaire (SPQ) and the Psychopathic Personality Inventory-Revised (PPI-R). After controlling for age and sex, regressions showed that the total SPQ score was positively related to the total PPI-R score and the Self-Centered Impulsivity factor, and negatively related to the Fearless Dominance factor. Self-Centered Impulsivity was positively related to all three SPQ factor scores, with the strongest relationship found with the Cognitive-Perceptual factor. In contrast, Fearless Dominance was negatively related to only the Interpersonal and Disorganized factors of the SPQ, with the strongest relationship found with the Interpersonal factor. Findings suggest that the comorbidity of schizotypy and the self-centered impulsivity aspect of psychopathy is not limited to extreme discrete populations, but exists in a more dimensional manner within a non-psychiatric sample. In addition, it appears that schizotypy is negatively related to the fearless dominance aspect of psychopathy, which appears to be a novel finding. Results provide preliminary findings that may have implications for developing appropriate prediction, assessment, and treatment techniques for violent behavior in schizophrenia-spectrum disorders.

## Introduction

Although the majority of patients with schizophrenia are not violent, rates of violence are relatively higher for individuals with schizophrenia when compared to most other psychiatric disorders (Krakowski et al., 1986; Hodgins et al., 1996; Joyal et al., 2007), particularly when schizophrenia is comorbid with substance abuse (Swanson et al., 1990; Erkiran et al., 2006). For instance, in one study, 8.36% of individuals with schizophrenia reported violent behavior compared to 3.45% of individuals with an affective disorder and 2.37% of individuals with an anxiety disorder (Swanson et al., 1990). Although 21.30% of individuals with substance abuse reported violence, the rate increased to 30.33% in individuals comorbid for both schizophrenia and substance abuse.

In comparison to the general population, a meta-analysis found that 9.9% of individuals with schizophrenia or another psychotic disorder were violent compared to 1.6% of the general population; however, authors found no increased risk of violence in individuals with comorbid psychosis and substance abuse compared to individuals with substance use disorders only (Fazel et al., 2009). Further, a recent study found that individuals with schizophrenia were 4.57 times more likely than individuals without schizophrenia to have been found guilty of a violent offense (Short et al., 2013). Once age, sex, and substance use were accounted for, individuals with schizophrenia were still 3.11 times more likely to have been found guilty of a violent offense, suggesting that increased violence in schizophrenia is not fully attributable to substance misuse.

In a recent review of the heterogeneity of violence within schizophrenia, Volavka and Citrome (2008) discussed three etiological subtypes of violent patients with schizophrenia; violence related to positive psychotic symptoms, impulsive tendencies, or comorbid psychopathy. The authors note that this third subtype may represent an unrecognized subtype of schizophrenia and inherently require different treatment considerations (e.g., non-pharmacological treatments in addition to typical antipsychotic or anticonvulsant medication management). Bo et al. (2011) similarly identified two trajectories of violent behavior in individuals with schizophrenia; violence corresponding to the emergence of positive symptoms and violence related to personality, particularly psychopathic traits.

Psychopathy was first defined systematically by Cleckley (1941) as characteristics of superficial charm, insincerity, lack of emotional reactions, and remorselessness, which differs from antisocial personality disorder (ASPD). ASPD is defined as pattern of deceitful, delinquent, and criminal behavior (American Psychiatric Association, 2000) whereas psychopathy describes callous-unemotional traits such as glibness, grandiosity, manipulativeness, and lack of empathy (Crocker et al., 2005). Although most individuals who endorse a high level of psychopathy meet criteria for ASPD, many individuals with ASPD do not endorse high levels of psychopathy (Hare, 1991; Crocker et al., 2005).

To date, much of the literature on the relationship between schizophrenia and violence has focused on the causal role of positive psychotic symptoms (McGregor et al., 2011), despite studies reporting prevalence rates for the comorbidity of psychopathy and schizophrenia to be between 17 and 19% (Rasmussen and Levander, 1996; Nolan et al., 1999). Further, the presence of elevated psychopathic traits in individuals with schizophrenia has been shown to relate to violence and related behaviors (e.g., Nolan et al., 1999; Tengström et al., 2000; Abushua'leh and Abu-Akel, 2006; Dolan and Davies, 2006; Fullam and Dolan, 2008; McGregor et al., 2011). The potential causal role of psychopathy is particularly important to understanding violence within this population, as personality traits persist beyond symptoms of schizophrenia (e.g., violence related to command hallucinations). This suggests that the propensity for violence may not dissipate following management of positive symptoms and thus requires diverse research, assessment, prevention, and treatment.

Although research has demonstrated a relationship between psychopathy and schizophrenia, it remains unclear whether the comorbidity found in these specialized severe samples extends to the non-clinical level of schizotypy (Raine et al., 2011). Schizotypy describes a latent personality construct genetically related to schizophrenia and schizotypal personality disorder (SPD) (Raine, 2006) which includes odd beliefs, unusual perceptual experiences, paranoid ideation, social anxiety, and odd speech and behavior (Raine, 1991). A growing body of research has supported a fully dimensional model of schizophrenia (Cochrane et al., 2010) which is conceptualized as a spectrum with schizotypy at the lower end and schizophrenia at the more severe end (Claridge and Beech, 1995). Conditions along this spectrum, including schizotypy and schizotypal personality disorder, have been shown to display similar neurocognitive deficits as seen in schizophrenia (e.g., Cadenhead et al., 1996; Bedwell et al., 2013), further supporting the genetic-relatedness of the spectrum. Interestingly, some measures of schizotypy include aspects of antisocial behavior and impulsivity as part of the construct (e.g., Oxford-Liverpool Inventory of Feelings and Experiences; O-LIFE; Mason et al., 1995), whereas others do not [e.g., Schizotypal Personality Questionnaire (SPQ); (Raine, 1991)]. Therefore, it remains unclear whether antisocial impulsivity is a core feature of schizotypy.

It appears that two studies have examined the relationship at the trait level, and both have been limited to incarcerated individuals. Specifically, Warren et al. (2003) found that female felons' SPD total symptom scores from a clinical interview were positively correlated with the Hare Psychopathy Checklist-Revised (Hare, 1991) impulsive/antisocial factor (i.e., Factor 2) and total score; whereas Raine (1992) found a positive relationship between psychopathy (assessed with the original PCL; Hare, 1985) and the total score from a self-report measure assessing both schizotypal and borderline personality features in a sample of prisoners. Although these studies begin to substantiate a likely downward extension of psychopathic endorsement, as both studies were restricted to incarcerated individuals, the comorbidity of these personality constructs in the non-forensic community remains unclear.

Relatedly, Raine et al. (2011) recently found that schizotypy was positively associated with total and reactive aggression (but not proactive aggression), in children aged 8–16. These findings suggest that peer victimization mediates the relationship between schizotypy and aggression, and further advocates for examination of adult schizotypy and psychopathy. These findings also suggests that public stigma of these populations may perpetuate a cycle of violence in a subset of these individuals, further highlighting the importance of understanding this relationship across all dimensions of the schizophrenia spectrum.

Overall, the research has clearly substantiated a comorbidity of psychopathy and schizophrenia in a subset of violent patients, and has begun to elucidate the relationship in the genetically-related, but less severe counterpart of schizotypy. However, there does not appear to be extant literature examining psychopathy and schizotypy in a non-forensic and non-psychiatric sample. By exploring how the traits and factors of psychopathy and schizotypy naturally correlate in a non-psychiatric and non-forensic sample, we can better understand which specific relationships may be driving the comorbidity observed in the more severe outcomes of schizophrenia and psychopathy. This directly addresses a gap in the literature as to whether the relationship exists within less severe expressions of schizophrenia, and provides a unique window into the comorbidity of schizophrenia and psychopathy in a sample with notably less frequent occurrences of incarceration, chronic neuroleptic use, hospitalizations, and/or severe active symptomatology, which may confound the relationship. This data can additionally be used to directly inform prevention and intervention efforts in the subset of individuals with schizophrenia-spectrum disorders who present with comorbid psychopathy, as these individuals may be at an increased risk for future violent behavior.

The present study sought to examine whether specific schizotypy factor scores were differentially related to the two factors of psychopathy in a sample of non-psychiatric and non-forensic adults. We first hypothesized that total schizotypy and psychopathy would be strongly and positively related based on the well documented association of schizophrenia and psychopathy. As previous research has not explored the relationship in this population using a dimensional approach, we further sought to explore how specific PPI-R factors would relate to schizotypy factors. A high score on Self-Centered Impulsivity indicates an individual with high self-centeredness, ruthless use of others, disregard of traditional values, propensity to blame others, estrangement, unconventional attitudes, and reckless impulsivity (Benning et al., 2003) which describes some attributes typically found in a subset of individuals with schizotypy (e.g., suspiciousness, odd or eccentric behavior, lack of close friends, and odd beliefs). Based on these theoretical similarities, and existing literature from forensic samples, we hypothesized that Self-Centered Impulsivity would be positively related to all schizotypy factors. Conversely, high scores on Fearless Dominance indicate an individual with a lack of anticipatory anxiety, low levels of tension and worry, low harm avoidance, and high levels of interpersonal dominance (Benning et al., 2003), whereas schizotypy traits include the seemingly inconsistent symptoms of social anxiety and paranoia. Therefore, we hypothesized that Fearless Dominance would be negatively related to all schizotypy factors.

## Materials and methods

### Participants

Undergraduate students from a large southeastern US university (*N* = 212; 50% female) completed the online study after providing informed consent. None of the participants showed evidence of inconsistent responding on the study measures (as measured by the Inconsistent Responding-15 scale within the PPI-R). The sample's mean age was 20.6 (*SD* = 4.44; range = 18–51) and the race was reported as 72.2% Caucasian, 9.9% African American, and 2.4% Asian American. 7.6% reported a “mixed” race, 7.1% “some other race,” and 0.9% did not describe their race. 12.3% of participants further reported an ethnicity of “Hispanic or Latino.”

### Procedure

This investigation was reviewed and approved by the University of Central Florida's Institutional Review Board, who approved an online informed consent statement. Participants learned of the study through a Psychology Department website, which offered multiple studies for students to participate in to receive academic credit toward a psychology course. The questionnaire was described to participants as a “survey of personality characteristics” and students were unaware that the questionnaire was intended to measure schizotypy or psychopathy. Following completion of the informed consent procedure, participants provided basic demographic information (i.e., age, gender, race, and ethnicity) and completed the 74-item SPQ and the 154-item Psychopathic Personality Inventory-Revised (PPI-R). No other measures were administered.

### Measures

#### Schizotypal personality questionnaire (SPQ)

The SPQ is a 74-item self-report measure of traits found in schizotypal personality disorder (Raine, 1991), which are consistent with DSM-IV diagnostic criteria (American Psychiatric Association, 2000). The SPQ provides an overall total score, which is the sum of all items, as well as nine subscales that load onto a three-factor model. The Cognitive-Perceptual factor consists of ideas of reference, odd beliefs or magical thinking, unusual perceptual experiences, and suspiciousness. The Interpersonal factor is comprised of social anxiety, no close friends, constricted affect, and suspiciousness. The Disorganized factor contains odd or eccentric behavior and odd speech. The SPQ has demonstrated substantial evidence for reliability and validity (Raine, 1991; Calkins et al., 2004; Mechri et al., 2010; Yasuda et al., 2011) with Cronbach's α ranging from 0.90 to 0.91 for the SPQ total score, and 0.63 to 0.81 for the nine subscales (Raine, 1991).

#### Psychopathic personality inventory-revised (PPI-R)

The PPI-R (Lilienfeld and Widows, 2005) is a 154-item self-report measure designed to give an overall dimensional measure of global psychopathy. The total score is comprised of two orthogonal factors, Fearless Dominance and Self-Centered Impulsivity, which are analogous to Hare's (2003) PCL-Revised interpersonal/affective factor (i.e., Factor 1) and impulsive/antisocial factor (i.e., Factor 2), and eight content scales. The content scales include Machiavellian Egocentricity (ME), which assesses for narcissistic and ruthless attitudes in interpersonal functioning; Coldheartedness (C), which assesses a propensity toward callousness, guiltlessness, and unsentimentality; Carefree Non-planfulness (CN), which assesses the attitude of indifference in planning one's actions; Fearlessness (F), which assesses the absence of anticipatory anxiety concerning harm and a willingness to participate in risky activities; Blame Externalization (BE), which assess the tendency to blame others for one's problems and to rationalize one's misbehavior; Social Influence (SOI) or the perceived ability to influence and manipulate others; Stress Immunity (STI), or the absence of marked reactions to anxiety-provoking events; and Rebellious Non-conformity (RN), or the reckless lack of concern regarding social norms. The Fearless Dominance factor is computed by summing the scores of SOI, F, and STI, whereas the Self-Centered Impulsivity factor includes ME, RN, CN, and BE. The measure has evidenced good internal reliability and test-retest reliability (Lilienfeld and Widows, 2005), good convergent validity with the PCL-R (Poythress et al., 2010), and good convergent validity with various psychopathy self-report measures (Uzieblo et al., 2010).

### Statistical analysis

The normality of the distribution for each variable was examined by inspecting the skewness and kurtosis. Scores from all scales followed a normal distribution (skewness and kurtosis < ± 2.00); therefore, parametric statistics were utilized for all analyses. Hierarchical regressions were conducted to control for the influence of age and sex and Pearson correlations were conducted to examine the zero-order correlations between all total and factor scores.

## Results

Descriptive statistics are provided in Table 1, which shows that male participants had higher scores on the PPI-R total and factor scores, as well as a statistical trend for higher scores on the SPQ Interpersonal factor.

**Table 1:** **SPQ and PPI-R descriptive statistics**.

	**Combined**	**Male**	**Female**
PPI-R Total *M* (*SD*) Range	289.62 (34.77)^***^ 190–384	300.14 (29.07) 224–367	279.10 (36.88) 190–384
PPI-FD *M* (*SD*) Range	113.12 (19.32)^**^ 60–165	117.27 (17.52) 77–153	108.97 (20.20) 60–165
PPI-SCI *M* (*SD*) Range	114.05 (23.30)^**^ 87–196	148.24 (23.77) 87–196	139.87 (22.15) 93–188
SPQ Total *M* (*SD*) Range	25.08 (14.83) 0–74	25.75 (15.31) 0–74	24.41 (14.37) 1–71
SPQ CogPer *M* (*SD*) Range	10.96 (7.56) 0–33	10.86 (7.40) 0–33	11.06 (7.75) 0–32
SPQ Int *M* (*SD*) Range	8.02 (5.77)^+^ 0–25	8.73 (6.13) 0–25	7.31 (5.32) 0–23
SPQ Dis *M* (*SD*) Range	6.10 (4.26) 0–16	6.17 (4.50) 0–16	6.02 (4.04) 0–16

Symbols reflect significant differences between sexes;

** p < 0.01,

*** p < 0.001,

+ p < 0.10 PPI-R, Psychopathic Personality Inventory-Revised; SCI, Self-Centered Impulsivity; FD, Fearless Dominance; SPQ, Schizotypal Personality Questionnaire; CogPer, Cognitive-Perceptual; Int, Interpersonal; Dis, Disorganized.

Zero-order correlations are provided in Table 2, which shows one significant negative relationship between age and the PPI-R Self-Centered Impulsivity factor.

**Table 2:** **The relationships between the PPI-R and SPQ total and factor scores**.

	**Age**	**PPI-R Total**	**PPI-FD**	**PPI-SCI**	**SPQ Total**	**SPQ CogPer**	**SPQ Int**	**SPQ Dis**
Age	1							
PPI-R Total	−0.123	1						
PPI-I FD	0.052	0.665^***^	1					
PPI-II SCI	−0.208^**^	0.782^***^	0.101	1				
SPQ Total	−0.055	0.182^**^	−0.242^***^	0.491^***^	1			
SPQ CogPer	−0.086	0.310^***^	−0.036	0.503^***^	0.874^***^	1		
SPQ Int	−0.008	−0.076	−0.443^***^	0.253^***^	0.799^***^	0.463^***^	1	
SPQ Dis	−0.027	0.184^**^	−0.211^**^	0.473^***^	0.849^***^	0.639^***^	0.605^***^	1

Values are Pearson correlation coefficients;

** p < 0.01,

*** p < 0.001, PPI-R, Psychopathic Personality Inventory-Revised; SCI, Self-Centered Impulsivity; FD, Fearless Dominance; SPQ, Schizotypal Personality Questionnaire; CogPer, Cognitive-Perceptual; Int, Interpersonal; Dis, Disorganized.

Due to these demographic relationships, we chose to conduct two hierarchical regressions to examine the relationships between the SPQ and PPI-R, with both age and sex entered in the first block, and the SPQ and PPI-R scores (respectively) in the second block.

**Figure 1:**
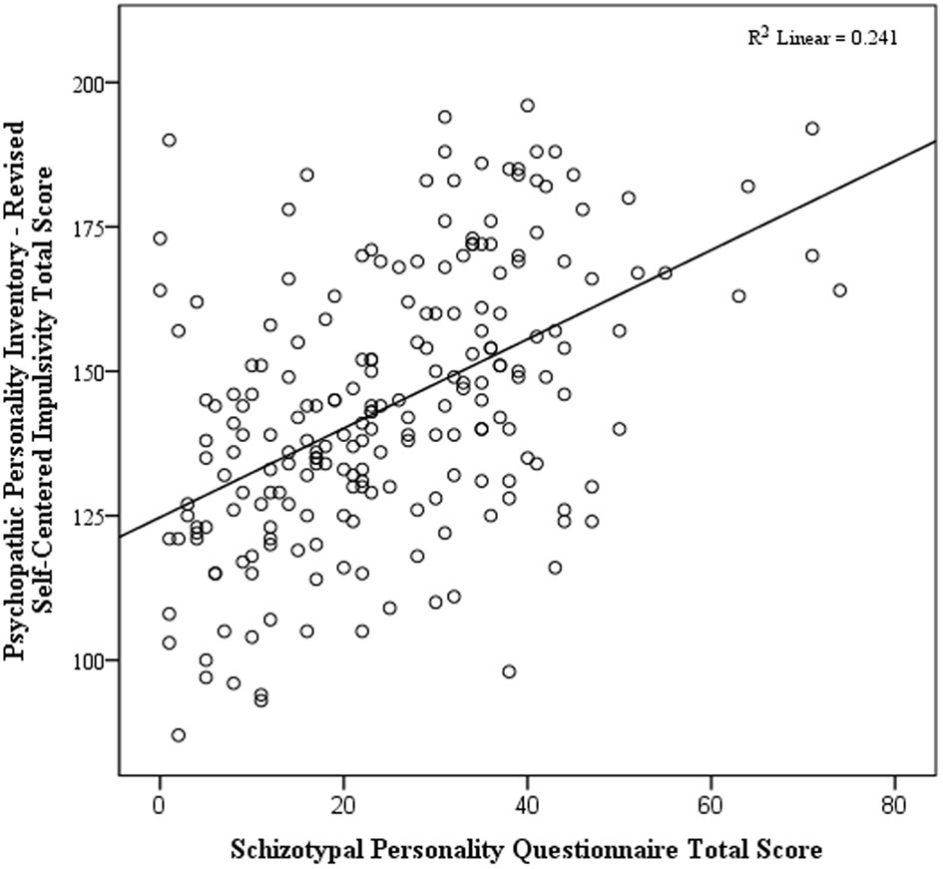
**Scatterplot of total schizotypy and self-centered impulsivity**.

**Figure 2:**
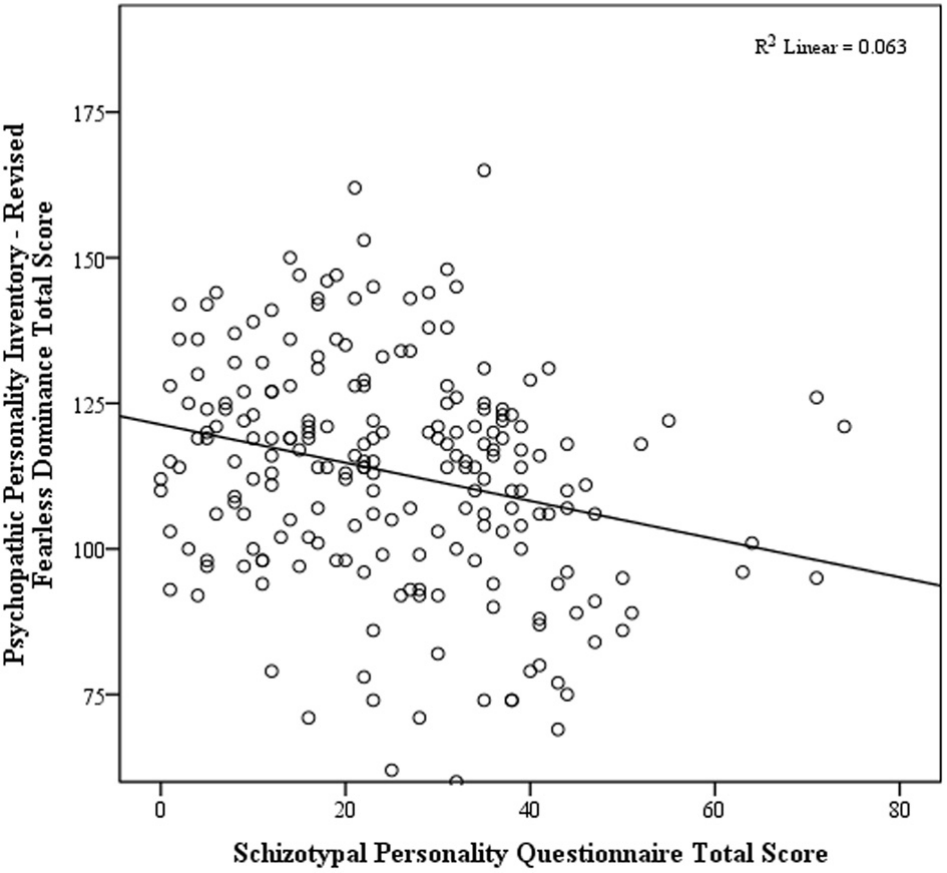
**Scatterplot of total schizotypy and fearless dominance**.

The first hierarchical regression examined the influence of the two PPI-R factor scores on the SPQ total score, after covarying for age and sex. This resulted in a significant overall model, *F*_(4, 207)_ = 26.40, *p* < 0.001, adjusted *R*^2^ = 0.33. There was a significant positive relationship between the SPQ total score and the PPI-R Self-Centered Impulsivity factor, β = 0.53, *t*_(207)_ = 9.06, *p* < 0.001 (see Figure 1), and a significant negative relationship with the Fearless Dominance factor, β = −0.31, *t*_(207)_ = 5.38, *p* < 0.001 (see Figure 2).

The second hierarchical regression examined the influence of the three SPQ factor scores on the PPI-R total score, after covarying for age and sex. This resulted in a significant overall model, *F*_(4, 206)_ = 17.68, *p* < 0.001, adjusted *R*^2^ = 0.30. There were significant positive relationships between the PPI-R total score with the SPQ Cognitive-Perceptual factor, β = 0.38, *t*_(206)_ = 4.97, *p* < 0.001, and Disorganized factor, β = 0.18, *t*_(206)_ = 2.07, *p* = 0.04, and a negative relationship between the PPI-R total score and the SPQ Interpersonal factor, β = −0.40, *t*_(206)_ = 5.41, *p* < 0.001.

## Discussion

The objective of this study was to examine the relationship between dimensional factors of psychopathy and schizotypy in a large sample of non-forensic and non-psychiatric individuals. Results support the initial hypothesis that overall schizotypy and psychopathy are positively related, substantiating the downward extension of psychopathic trait endorsement to the non-clinical schizotypy level. However, findings suggest that schizotypy, and perhaps schizophrenia by extension, are only related to one aspect of psychopathy—Self-Centered Impulsivity. As the other aspect of psychopathy, Fearless Dominance, was negatively related to schizotypy, the positive correlation between the two total scores was clearly driven by the association of schizotypy with the Self-Centered Impulsivity aspect of psychopathy. The overall findings suggest a positive dimensional relationship between the schizophrenia spectrum disorders and the Self-Centered Impulsivity aspect of psychopathy, as this relationship exists in even high-functioning college students.

This finding further lends support to the inclusion of impulsivity as a core feature of schizotypy as is done in some schizotypy measures (e.g., O-LIFE; Mason et al., 1995), especially as it correlated positively with total schizotypy and all three factors. Further substantiating the largely orthogonal nature of the two PPI-R factors (Lilienfeld and Widows, 2005; Marcus et al., 2013), our findings suggest that the three schizotypal symptom factors relate differentially to each PPI-R factor. Specifically, a higher score on Self-Centered Impulsivity was related to a higher score on the Cognitive-Perceptual, Interpersonal, and Disorganized factors of schizotypy, whereas a higher score Fearless Dominance was related to a lower score on the Interpersonal and Disorganized factors. Interestingly, a previous study reported that Self-Centered Impulsivity, the psychopathy factor positively associated with schizotypy, was associated with a higher level of substance abuse (Lilienfeld and Widows, 2005), which may be especially important considering that rates of violence are particularly elevated when schizophrenia is comorbid with substance abuse (Swanson et al., 1990; Erkiran et al., 2006). Further, results indicated that Self-Centered Impulsivity showed the strongest relationship with the Cognitive-Perceptual schizotypy factor, suggesting that the likelihood of comorbid Self-Centered Impulsivity traits is highest in individuals with a particularly high level of positive symptoms. Individuals with a schizophrenia-spectrum disorder who have marked positive symptoms may therefore be at a higher risk for violent behavior due to two theoretical mechanisms: a higher likelihood of comorbid Self-Centered Impulsivity and the increase in violence that is a direct response to the positive symptoms themselves as proposed by others (Volavka and Citrome, 2008; Bo et al., 2011).

The negative relationship found between schizotypy and the Fearless Dominance factor is equally important to understanding this complex relationship. Interestingly, the negative correlation between Fearless Dominance and schizotypy appears to be primarily influenced by the Interpersonal schizotypy factor, which includes symptoms such as social anxiety, lack of close friends, and constricted affect. Therefore, individuals endorsing high levels of these interpersonal symptoms, even in the presence of high Self-Centered Impulsivity, may be less likely to experience the callous and unemotional traits related to psychopathy. Therefore, the overall risk of psychopathic behavior (such as violence) remains unclear in individuals who endorse both positive and interpersonal symptoms of schizotypy, as one risk factor and perhaps one protective factor are present.

Future research on the investigation of the relationships between schizophrenia-spectrum disorders, psychopathy, and violence would benefit from a continued effort to elucidate separate etiological pathways toward violence, which could directly inform clinical practice. Specifically, findings may indicate the need for more routine assessment of psychopathy in the initial assessment of individuals with schizophrenia-spectrum disorders—particularly in patients with prominent positive symptoms. Treatment of individuals who are comorbid for a schizophrenia-spectrum disorder and aspects of psychopathy would likely benefit from the development of new treatment protocols which are more directly targeted at reducing the risk of future violence.

The current study extends prior work on the overlap of schizophrenia and psychopathy through a novel investigation of the genetically-related, less severe construct of schizotypy in a non-forensic and non-psychiatric sample. However, the study is limited by the use of undergraduate students, as the findings may not generalize to other non-forensic and non-psychiatric adults. Additionally, although studies involving self-report data may possess inherent limitations, Lilienfeld and Fowler (2006) point out that self-report assessment of psychopathy possesses certain advantages. For instance, specific core features of psychopathy (e.g., lack of empathy and remorse) require considerable clinical inference, and thus may be better assessed by self-report. Further, as our sample assessed dimensional schizotypal traits and not diagnosed schizotypal personality disorder or schizophrenia, further research is needed to determine if similar differential relationships between these two factors of psychopathy exist in clinical populations. Finally, and arguably most importantly, the current study did not assess violent behavior and therefore cannot directly assess a potential mediating role of psychopathy in the relationship between schizotypy and violent behavior. Future research is needed to directly assess violent behavior in the context of these relationships.

Overall, these results provide tentative directions for understanding the relationship between psychopathy and schizotypy. The findings can serve as a starting point for future studies to examine frequency and etiology of psychopathic traits among individuals with schizophrenia-spectrum disorders and traits. This line of research may lead to empirically-supported screening measures to identify individuals with a schizophrenia-spectrum disorder who may be at an increased risk of future violent behavior due to elevated psychopathic traits. Further research within this area is needed to determine if individuals who endorse a high level of both schizotypy and Self-Centered Impulsivity represent a new and useful subtype of schizotypy and/or SPD. Future experimental research is also needed to clarify whether those with comorbid schizotypy and Self-Centered Impulsivity exhibit some of the same physiological and neurocognitive abnormalities that have been reported in those with primary psychopathy.

### Conflict of interest statement

The authors declare that the research was conducted in the absence of any commercial or financial relationships that could be construed as a potential conflict of interest.
